# Morphine versus methylprednisolone or aminophylline for relieving dyspnea in patients with advanced cancer in China: a retrospective study

**DOI:** 10.1186/s40064-016-3651-x

**Published:** 2016-11-09

**Authors:** Cong Tian, Jiong-Yi Wang, Mei-Ling Wang, Bin Jiang, Lu-Lu Zhang, Feng Liu

**Affiliations:** Department of Medical Oncology, Shanghai Ninth People’s Hospital, Shanghai Jiao Tong University of Medicine, Shanghai, 201900 China

**Keywords:** China, Morphine, Methylprednisolone, Aminophylline, Dyspnea, Advanced cancer

## Abstract

**Context:**

Dyspnea is one of the most common and distressing symptoms that occurs in terminal cancer patients. However, there are no existing treatment guidelines for this condition in China.

**Objective:**

This single-center, retrospective, observational study aimed to compare the efficacy of using morphine, methylprednisolone, or aminophylline to relieve the symptom of breathlessness in patients with advanced malignant tumors and to investigate the safety of these regimens during the treatment of dyspnea.

**Methods:**

Between August 2011 and January 2015 we retrospectively reviewed the medical records of 343 terminally ill cancer patients with dyspnea who received morphine, methylprednisolone, or aminophylline. The therapeutic effect of each treatment by means of visual analogue scale (VAS) scores was assessed and compared. Statistical methods included Chi square and analysis of variance tests. Differences were considered significant when *P* < 0.05.

**Results:**

VAS scores after treatment were (16.82 ± 10.89), (25.72 ± 15.03), and (31.95 ± 16.00) points in the morphine, methylprednisolone, and aminophylline group, respectively. These differences were found to be significantly different (*P* < 0.05). The effectiveness ratings were 86.44, 62.16, and 49.12%, respectively (*P* < 0.05).

**Conclusions:**

We found that morphine subcutaneous injection for advanced cancer patients with dyspnea was safe and typically more effective than methylprednisolone or aminophylline. Therefore, morphine treatment could significantly improve the quality of life in terminal cancer patients with short life expectancies who are experiencing shortness of breath.

## Background

Dyspnea, or breathlessness, is a subjective sensation described as uncomfortable or unpleasant labored breathing (air hunger) (Williams 2006). It is a devastating symptom that frequently occurs in advanced cancer patients. In advanced cancer, patients with any tumor, regardless of its primary site, may experience breathlessness. However, it is the most common in primary pulmonary carcinomas and in patients with metastatic lung disease (Parshall et al. 2012). Almost 50–70% of patients with advanced cancer experience intractable dyspnea in the last 6 weeks, and dyspnea is the main symptom in more than 20% of patients within their final 48 h of life (Williams 2006; Parshall et al. 2012; Navigante et al. 2006). Symptoms such as loss of appetite, fatigue, pain, and dyspnea reduce the quality of life (QoL) in cancer patients (Polanski et al. 2016). These symptoms usually become gradually aggravated as the tumor progression.

Assessment of dyspnea, as presented in the published medical literature, follows a pattern similar to that of pain. However, all of the objective data, such as respiratory rate, arterial blood gases, and oxygen saturation, cannot be used to quantitatively measure dyspnea. Therefore, assessing dyspnea relies on subjective measures, and the patient’s subjective self-report of breathlessness is the most valid and reliable indicator of its presence (Thomas and von Gunten 2002; Mancini and Body 1999). Respiratory depression severely impairs the quality of life in these patients, and is usually accompanied by anxiety, depression, fear, and even panic (Polanski et al. 2016). These extremely unpleasant feelings further exacerbate the problem and immensely reduce the patient’s self-esteem and self-confidence. The development of standardized treatments for cancer pain has received considerable attention throughout the world (Caraceni et al. 2012). However, in contrast to pain, breathlessness in terminally ill cancer patients is often not taken seriously and is frequently poorly managed in China (Lai et al. 2007).

The most effective measures to alleviate dyspnea are to address the underlying cause(s) of the breathlessness, such as resolving infection, controlling tumor growth, correcting anemia, and draining malignant pleural effusions (Williams 2006; Dy et al. 2008). Nevertheless, there is often no discernable treatable cause or patients are not eligible for etiological treatment due to their poor clinical state and overall health. Therefore, symptomatic treatment is often necessary, and palliative care then focuses on the relief of suffering and on symptom control.

At present, morphine sulfate is internationally recommended as the first-line therapy for cancer-related dyspnea (Ben-Aharon et al. 2008, 2012). However, in China, primary interventions, including oxygen, methylprednisolone, antiasthmatic drugs and benzodiazepine are often used to alleviate the symptoms of breathlessness in patients with advanced cancer. At the same time, morphine may be used for cancer-related dyspnea in terminally ill cancer patients, although many physicians and patients are reluctant to use morphine because of the concern for respiratory depression and the lack of comprehensive understanding about morphine.

Currently, there are only few high-quality, large-sample randomized controlled trials (RCTs) that assess the effects of morphine in the palliation of cancer-related breathlessness (Barnes et al. 2016). Therefore, we retrospectively reviewed the medical records to evaluate the efficacy of using morphine to alleviate dyspnea sensations during the last 6 weeks of life in terminally ill cancer patients. To our knowledge, this is the first report to compare morphine, methylprednisolone, and aminophylline therapy for patients with cancer-related breathlessness. The aim of this retrospective study was to provide significant information for clinical use and to improve our understanding of how beneficial/safe morphine may be for the treatment of cancer-related breathlessness in China.

## Methods

### Study design and patient population

A single-center, retrospective study was conducted in the department of Oncology, at Shanghai Ninth People’s Hospital, School of Medicine, Shanghai Jiao Tong University from August 2011 to January 2015. This study was approved by the Regional Ethics Committee of our hospital and the study complied with the Declaration of Helsinki. The patient’s information was anonymized and de-identified prior to analysis. Patients with advanced, stage III or IV cancer undergoing palliative treatment (alleviating the suffering and improving the life quality of patients) were eligible to participate in the study. Patients with advanced malignant tumors were confirmed by clinical and imaging diagnosis. Demographic patient- and disease-related data, such as the type and dose of drugs, visual analogue scale scores, side effects during admission were evaluated and documented by the physician in charge at our palliative care unit, including all clinically relevant laboratory parameters.

We retrospectively selected patients who met the following criteria: (1) those that were at least 18 years old, (2) those that were diagnosed pathologically with cancer, (3) those that had provided the subjective self-report of moderate-to-severe dyspnea, such as uncomfortable, distressful, or labored breathing, (4) those with a serum creatinine concentration within twice the normal range, (5) those with a life expectancy less than one month, and (6) those undergoing medicine intervention for dyspnea.

Patients were excluded if they met any of the following criteria: (1) had a serious renal or hepatic failure (biochemically and/or clinically detected), (2) had active or uncontrolled chronic obstructive pulmonary disease (COPD) and serious lung infection, (3) were diagnosed with hemoglobin saturation by pulse oximetry (SaO_2_) <85%, (4) had superior vena cava syndrome (SVCS), (5) had non-compensated congestive heart failure, or 6) had a contraindication to morphine, benzodiazepine drugs, and hormones.

### Treatment regimen

All eligible patients complaining of shortness of breath in bed received non-pharmacological support (oxygen therapy using nasal prongs at a rate of 2–6 L/min) as the foundation of treatment. According to the treatment received, we divided patients into the three groups as follows. In the morphine group, opioid-naïve patients were given 5 mg morphine subcutaneously, and opioid-tolerant patients were injected subcutaneously with 10% of the total opioid taken in the previous 24 h. In the methylprednisolone group, 40 mg methylprednisolone in 100 ml normal saline was administered intravenously by drip for 30 min. In the aminophylline group, 0.25 g aminophylline with 5% glucose injection was administered intravenously by drip for 30 min.

### Assessment

As is the case with pain, adequate assessment of dyspnea depends on self-report. A 100 mm visual analogue scale (VAS) was used to quantify breathlessness. The VAS scores were performed according to standard procedures. VAS is a measurement apparatus that tries to measure a characteristic or attitude that is believed to range across a continuum of values and cannot easily be directly measured (Pianosi et al. 2016). Operationally, a VAS is usually a horizontal line, 100 mm in length, anchored by word descriptors at each end. Anchors are “no breathlessness’’ at 0 mm and “worst imaginable breathlessness” at 100 mm (Gould et al. 2001). The patient makes a mark on a 100-mm line with descriptors at each end corresponding to the extent of their symptom before and after treatment. Intensity of breathlessness was recorded by our palliative care unit before using any drugs and 1 h after one dose. Dyspnea relief was considered effective when the VAS numerical value decreased by more than 50%. The study’s principal endpoint was the effective rate of the intervention. Additional endpoints included the frequency and severity of medication-related side effects. After 1 h of a single-dose, adverse effects were assessed and documented on a daily basis.

### Statistical analysis

Descriptive statistics including frequencies, means, standard deviation (SD), medians, and ranges were used to examine the distribution of measures as appropriate. The Pearson’s Chi squared test was undertaken to examine bivariate associations between categorical variables, and analysis of variance (ANOVA) was undertaken to examine the associations between categorical and continuous variables. The data were reported as mean SD. The P-values cited were derived from a two-tailed test, and P values less than 0.05 were considered statistically significant. All calculations were performed with SPSS software version 19.0 (SPSS, Inc., Chicago, IL, USA).

## Results

### Patients characteristics

Information was collected from patient medical records; between August 2011 and January 2015, 343 cancer patients with moderate to severe dyspnea were enrolled in this study and were divided into three groups. 118 patients were treated with morphine. 111 patients were treated with methylprednisolone. 114 patients were treated with aminophylline. The average age of the patients was 53.32 ± 18.29 years old (range 22–79 years). 174 patients were male and 169 patients were female. Overall, 125 of 343 (36.44%) patients had lung cancer, 96 (27.99%) had breast cancer, 46 (13.41%) had colorectal cancer, 41 (11.95%) had gastric cancer, and 35 (10.2%) had other cancers. There were no significant differences in clinical materials for each group (*P* > 0.05). The baseline characteristics of the patients included were listed in Table 1.

**Table 1:** Characteristics of the patients (n = 343)

Characteristic	Morphine	Methylprednisolone	Aminophylline
Patients	118	111	114
Age in years (mean)	54.2	53.1	53.7
Sex (M/F)	61/57	54/58	60/54
Diagnoses			
Tumor (n)			
Lung	45 (38.1%)	37 (33.3%)	43 (37.7%)
Breast	30 (25.4%)	34 (30.6%)	32 (28.1%)
Colorectal	15 (12.7%)	17 (15.3%)	14 (12.3%)
Gastric	15 (12.7%)	13 (11.7%)	13 (11.4%)
Others	13 (11%)	10 (9%)	12 (10.5%)

### A decline in VAS scores was more obvious in the morphine group

After an hour, the results were recorded. In the morphine group, VAS scores were reduced from 65.06 ± 13.27 to 16.82 ± 10.89 mm. In the methylprednisolone group, VAS scores were reduced from 64.04 ± 12.09 to 25.72 ± 15.03 mm. In the aminophylline group, VAS scores were reduced from 64.43 ± 11.86 to 31.95 ± 16.00 mm. There were no statistically significant differences in the VAS scores between groups before treatment (*P* = 0.820). However, there was a statistically significant difference in the VAS scores after intervention (*P* = 0.000; Table 2).

**Table 2:** Before and after treatment VAS score comparison

Groups	Morphine	Methylprednisolone	Aminophylline	*F* value	*P* value
VAS score before treatment	65.06 ± 13.27	64.04 ± 12.09	64.43 ± 11.86	0.198	0.820
VAS score after treatment	16.82 ± 10.89	24.58 ± 17.51	31.95 ± 16.00	31.721	0.000

*VAS* visual analogue score

### The effective rate was statistically different between the three groups

The treatment was deemed to be effective when the VAS scores were reduced by at least 50%, such that an effective case was defined as ([(VAS score before treatment − VAS score after treatment)/VAS score before treatment] × 100%) ≥ 50%. The effective rate was calculated as follows: (number of effective case/total case number) × 100%. The effective rates of patients who experienced dyspnea relief were 86.44, 62.16, and 49.12% in the morphine, methylprednisolone, and aminophylline groups, respectively. There was a statistically difference between the morphine group and other two groups (*χ* ^2^ = 17.83, *P* = 0.000 and *χ* ^2^ = 37.17, *P* = 0.000 for morphine vs. methylprednisolone and morphine vs. aminophylline, respectively) (Table 3).

**Table 3:** Comparison of effective rate between morphine and the other two groups

Groups	Total number	Effective number	Effective rate (%)	*χ* ^2^ value	*P* value
Morphine	118	102	86.44		
Methylprednisolone	111	69	62.16	17.826	0.000*
Aminophylline	114	56	49.12	37.172	0.000*

* Comparison with morphine group

### Adverse effects

Adverse effects (AEs) are summarized in Table 4. We recorded no drug-related deaths and no serious AEs that required drug discontinuation. No cases of serious respiratory depression were observed. No patients reported evident side effects after drug intervention in the methylprednisolone and aminophylline group. In the morphine group, the most frequently observed AEs were somnolence and constipation. Roughly half of the cases experienced somnolence, and 12 patients required a dose reduction to ease excessive somnolence.

**Table 4:** Adverse events related to the treatments

Adverse events	Morphine	Methylprednisolone	Aminophylline
Somnolence	62	None	None
Constipation	55	None	None
Nausea/vomiting	28	None	14
Dizziness	19	None	None
Flushing	3	11	None
Xerostomia	5	None	None

## Discussion

Breathlessness, or shortness of breath, refers to discomfort and difficulty in breathing. The medical terminology for breathlessness is dyspnea or respiratory depression (Williams 2006). People describe the feeling of breathless in different ways. Patients often use the phrases “shortness of breath,” “cannot get enough air,” “out of breath,” or “tightness in my chest.” Dyspnea is one major cause of suffering in cancer patients, and it impairs the patient’s quality of life as severely as cancer pain.

In Western countries, exogenous opioid drugs, such as morphine sulfate, are recognized as the first-line of therapy in the symptomatic treatment of breathlessness in patients with advanced malignant tumors (Williams 2006; Dy et al. 2008; Ben-Aharon et al. 2012; Cheung and Zimmermann 2011). In China, corticosteroids and bronchodilators are widely used to treat the bronchoconstriction associated with asthma and chronic obstructive pulmonary disease (COPD) (Walters et al. 2014; Nardini et al. 2014). These medications can reduce airway inflammation and edema and reduce the sense of effort with breathing. Therefore, clinicians empirically apply methylprednisolone and aminophylline to relief dyspnea in patients that have terminal cancer. However, morphine is still rarely used by Chinese doctors for the treatment of cancer-related dyspnea. Many clinicians are uncertain as to which drug might be most effective and whether or not morphine should be used to be treat cancer-related dyspnea. At present there is a lack of related literature that directly compares these three drugs, and there are no existing guidelines for the management of dyspnea in China.

In this research, 343 patients were divided into three groups based on the treatment received. Subcutaneous and intravenous routes of administration were used in the study, because the ability to swallow declines and consciousness often wanes during the last weeks of life. Our results showed that morphine was superior to methylprednisolone and aminophylline for controlling the symptoms of cancer-related dyspnea. The VAS scores were 16.82 ± 10.89, 25.72 ± 15.03, and 31.95 ± 16.00 mm (*P* = 0.000) after treatment in the morphine, methylprednisolone, and aminophylline groups, respectively. Moreover, the effective rate of morphine was also obviously higher than that of the other drugs. The symptom improvement was more apparent in the morphine treatment group, while patients treated with methylprednisolone and aminophylline usually required repeat doses.

At the same time, this study implied that adverse effects of morphine treatment were generally well tolerated, with somnolence and constipation being the most common AEs. Neither respiratory depression nor severe sedation was observed in the morphine group. In China, clinicians, patients, and their families are still reluctant to use morphine, because of the fear of respiratory depression. In fact, there is no need to be overly concerned with respiratory depression. Morphine has been widely used to treat cancer pain, and the incidence of respiratory depression has been proven to be uncommon (Mercadante 2010). Even if respiratory depression were to occur, the opioid antagonist naloxone can quickly and effectively reverse respiratory depression (Howlett et al. 2016; Taylor et al. 2013). There are few reports concerning morphine poisoning deaths, and no evident serious adverse effects (respiratory depression) were observed in the recent literature concerning the use of morphine for alleviating cancer-related dyspnea (Ben-Aharon et al. 2012).

Morphine is often used to treat severe cancer pain and acute heart failure (Nardini et al. 2014; Iakobishvili et al. 2011). However, the use of morphine is at risk of addiction and respiratory depression (Chidambaran et al. 2015). Many physicians and patients are reluctant to use morphine for fear of respiratory depression and the lack of comprehensive understanding about morphine. Hence, in China, morphine is considered to be the contraindicated for dyspnea (due to the risk of respiratory depression). Additionally, although morphine has been prohibited to treat COPD for a long time, it is presently the first-line drug used to alleviate breathlessness for patients with COPD (Zebraski et al. 2000; Mahler et al. 2010; Verberkt et al. 2016). Increasing numbers of clinical studies support the notion that morphine should be a first-line drug that is effective and safe for the treatment of dyspnea in terminal cancer. It does not accelerate the deterioration of the patient’s condition or hasten death (Bonnichon et al. 2008). The National Comprehensive Cancer Network (NCCN) clinical practice guidelines in oncology palliative care recommend that opioids are one class of effective drugs available for cancer-related dyspnea, especially those patients who have a shorter life expectancy. Morphine sulfate, a representative opioid is effective in alleviating the symptoms of breathlessness, decreasing the ventilator response to hypoxia and hypercapnia, while decreasing oxygen consumption at rest and exercise (Williams 2006).

In contrast to vision, hearing, and pain, the neurophysiology of cancer-related dyspnea is far less understood. The reason why morphine relieves dyspnea may include the following aspects. Dyspnea in cancer patients is usually associated with the degree of anxiety, fear, panic, and depression, which can be improved by morphine arising to be mixed undisturbedly demulcent action (Polanski et al. 2016). Morphine probably acts both by depressing spontaneous respiratory drive and by modulating cortical activity, much like what happens with morphine treatment for pain. Current studies have shown that dyspnea and pain are both influenced by a common central nervous control area; there is speculation that the mechanism of morphine-mediated dyspnea relief is similar to the mechanisms that alleviate pain (Clemens et al. 2008). In addition, morphine may also play a role in decreasing the sensitivity to dyspnea, improving the body’s tolerance and cardiovascular function, reducing the ventilator response to hypoxia and hypercapnia (Zebraski et al. 2000). It is possible that the influence of these different mechanisms varies in different patients.

Undoubtedly, it is also extremely important to treat the underlying cause of the breathlessness, such as a reduction hydrothorax and ascites (Williams 2006; Dy et al. 2008; Bausewein et al. 2008). Moreover, benzodiazepines (BZDs) may be considered as a second- or third-line treatment in patients when dyspnea is not relieved by opioids, particularly if anxiety is a significant coexisting symptom (Clemens and Klaschik 2011).

The present work has several limitations. First, this study is a retrospective study. Data collection relied on medical record review, and the documentation may have been limited. Second, our sample size is not large enough to definitively state that morphine is superior to other treatments for dyspnea. Third, there may be the potential for confounding factors, such as psychosocial and patient factors that were not measured in the context of this study, but could impact our results.

## Conclusions

Morphine is generally safe and significantly more effective than methylprednisolone and aminophylline in management of dyspnea for terminal cancer patients. This treatment could significantly help improve the quality of life in such patients, although further research is needed to detail and clarify the efficacy of morphine for the treatment of dyspnea in patients with advanced malignant tumors. It is our opinion that Chinese oncologists should consider changing their static/conservative views on morphine, and strive towards understanding the special role of morphine in the management of cancer-related dyspnea to better control/minimize suffering in terminal cancer patients.
